# A rare cause of abdominal pain managed unconventionally: acute renal infarction caused by atrial fibrillation: a case report

**DOI:** 10.1186/s13256-022-03608-z

**Published:** 2022-10-19

**Authors:** Tao Ge, ZhengCai Zhu, Jinfeng Wang, Wenjiao Zhou, Evelyn J. Song, Shengxing Tang

**Affiliations:** 1 Department of Cardiology, The First Affiliated Hospital, Wannan Medical College, No.2, Zhe Shan West Road, Wuhu, 241001 Anhui China; 2 Department of Electrocardiogram, The First Affiliated Hospital, Wannan Medical College, Wuhu, Anhui China; 3 Division of Cardiology, University of California San Francisco, San Francisco, CA 94143 USA

**Keywords:** Acute renal infarction, Interventional therapy, Atrial fibrillation, Abdominal pain

## Abstract

**Background:**

Atrial fibrillation is one of the most common arrhythmias. The main thrombotic complication of arterial fibrillation is ischemic stroke, but it can also cause acute renal infarction from embolization. The low incidence and nonspecific clinical manifestations of acute renal infarction make it difficult to diagnose, often leading to either delayed diagnosis or misdiagnosis. Due to its rarity, more efficient treatment guidelines are helpful for the management of acute renal infarction related to the thromboembolic complication of arterial fibrillation.

**Case reports:**

We report a case of acute renal infarction due to underlying arterial fibrillation, where a novel interventional therapeutic method was used. A 66-year-old Chinese man with arterial fibrillation, not on anticoagulation due to the patient’s preference, and coronary artery disease post-percutaneous coronary intervention to left anterior descending artery about 1 year ago, was currently on dual antiplatelet therapy. He suddenly developed intermittent and sharp left-sided abdominal pain and was found to have an acute left renal infarction on computed tomography scan. Angiogram showed acute occlusion of the left renal artery due to thromboembolism. For this patient, a combination method of local thrombus aspiration, angioplasty, and infusion of nitroglycerin and diltiazem were used, restoring blood flow to the left kidney. After recovery, the patient was discharged on aspirin, clopidogrel, and warfarin. At 6 months follow-up, there was no residual kidney dysfunction.

**Conclusions:**

Acute renal infarction from thromboembolism is a rare but serious complication of arterial fibrillation. More efficient and different options for intervention methods will benefit the treatment of this disease. Here, we report a combination therapeutic method that has not been used in acute renal infarction associated with arterial fibrillation, and which restored renal perfusion and prevented long-term kidney injury.

## Introduction

Acute renal infarction (ARI) is a serious medical emergency and can lead to irreversible damage to the kidneys. Causes of renal infarction can be divided into four main groups: renal infarction of cardiac origin, renal infarction associated with renal artery injury, renal infarction associated with hypercoagulability disorders, and idiopathic renal infarction [1]. Atrial fibrillation (AF) is one of the most common arrhythmias, and ARI is a very rare but serious complication of AF. Diagnosis of ARI is challenging due to its low incidence and nonspecific clinical presentations. Currently, more efficient treatment guidelines are helpful for ARI due to thromboembolic complications of AF [1, 2], although aspiration thrombectomy is reported in the treatment of renal artery thromboembolism[3–5]. Also, treatment methods employed in prior case reports included systemic thrombolytics, intra-arterial thrombolytics, and systemic anticoagulation [6–8]. Given the rarity of ARI from AF and the lack of more efficient and well-established treatment guidelines, here we report a case of AF-induced ARI and present a new treatment modality using a combination of vasodilators, manual thrombus aspiration, and balloon angioplasty, similar to the treatment of acute myocardial infarction, which results in the successful treatment of ARI from AF.

## Case description

The patient was a 66-year-old Chinese man with a past medical history significant for AF, not on anticoagulation per the patient’s preference, and coronary artery disease (CAD) status post-percutaneous coronary intervention to left anterior descending artery about 1 year ago, currently on aspirin, clopidogrel, and rosuvastatin. He initially presented with 2 days of chest tightness, and acute coronary syndrome was ruled out. He then suddenly developed intermittent and sharp left-sided abdominal pain. Systemic review was negative for fever, chills, nausea, vomiting, diarrhea, or hematuria. He lives in a rural town working as a farmer, and is a current smoker with about 30 pack year smoking history. He denies any alcohol abuse or illicit drug use, and has no known drug allergies. Family history is non-contributory. Current home medications include aspirin (100 mg), clopidogrel (75 mg), rosuvastatin (10 mg), furosemide (20 mg), spironolactone (20 mg), and isosorbide mononitrate (10 mg). No known drug allergies were documented.

On admission, his vitals were normal, with blood pressure of 100/70 mmHg, heart rate of 68 beats/minute, and oral temperature of 36.3 °C. On examination, his heart rate was irregularly irregular without heart murmurs, jugular venous pressure (JVP) was normal, with no pitting edema in lower extremities. Lungs were clear on auscultation. His abdominal examination was notable for mild tenderness to palpation over the left quadrant without rebound or guarding. Bowel sounds were normal. The rest of the examination was unremarkable.

**Fig. 1:**
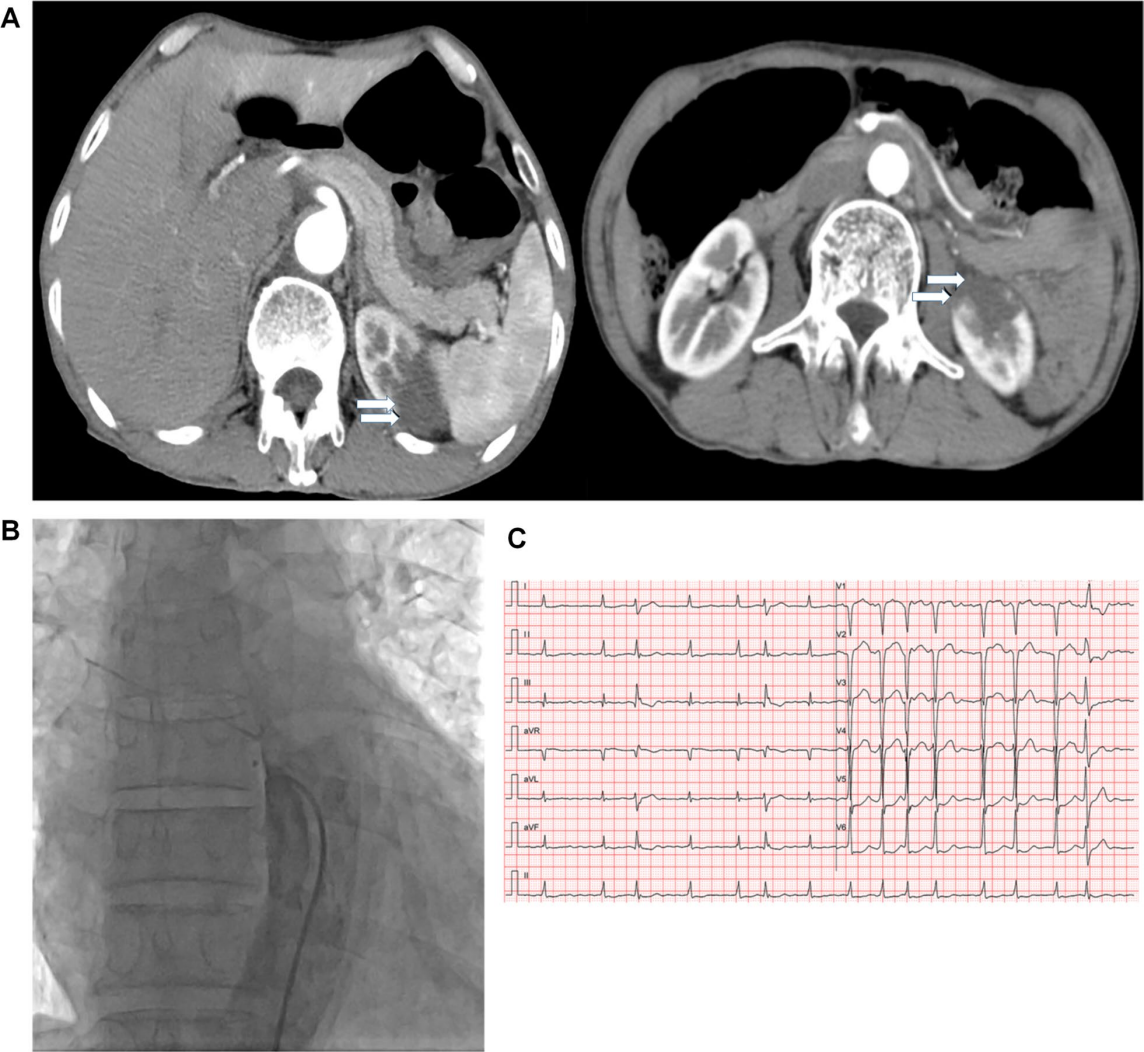
Contrast-enhanced computed tomography (CT) scans of the abdomen with intravenous contrast showed perfusion defect in the left kidney (**A**, arrows), Aortogram (**B**), and electrocardiogram (EKG) (**C**)

**Fig. 2:**
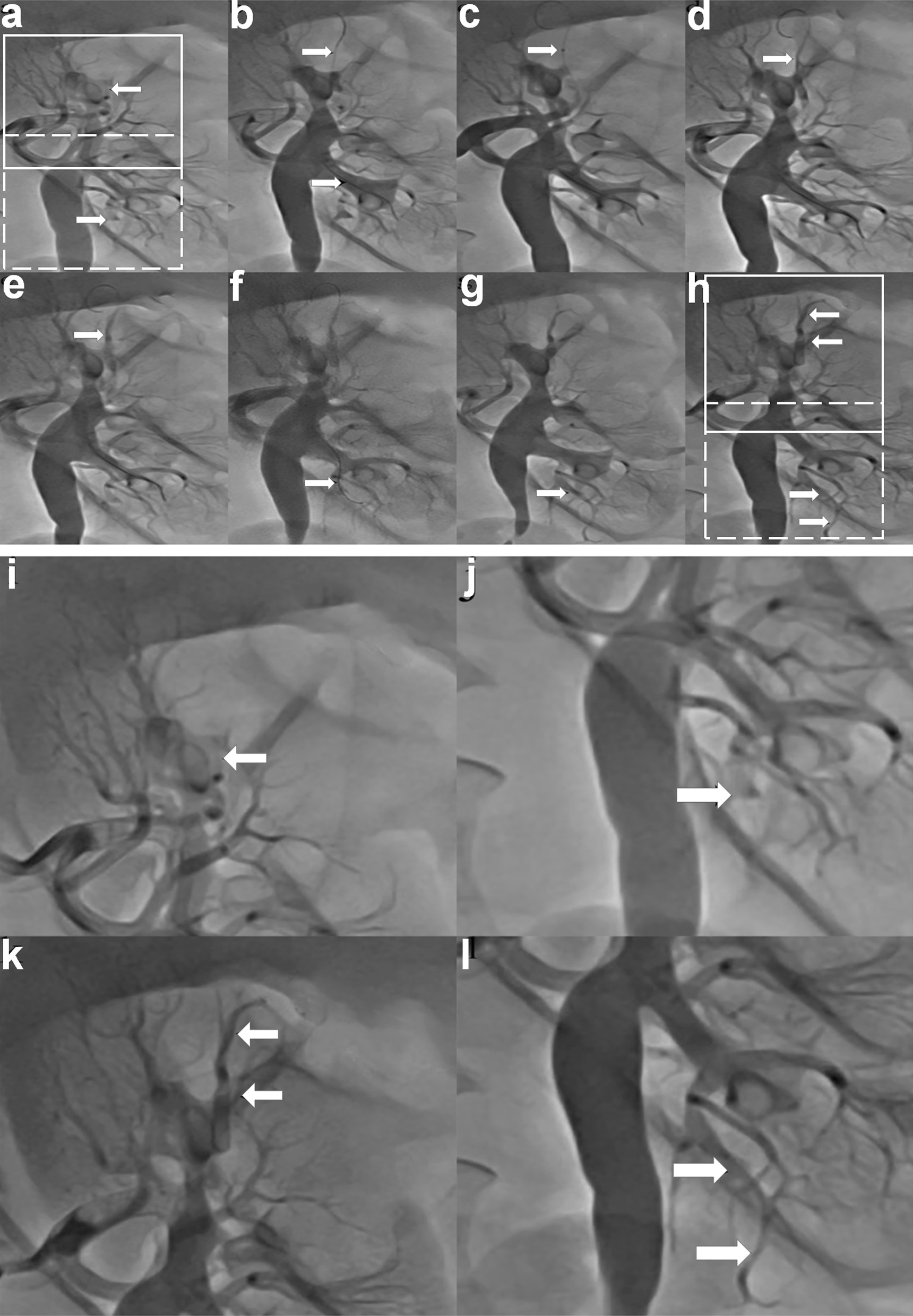
Intraoperative angiography images (**a**) Initial angiogram showing filling defects in the superior and inferior branches of the left renal artery (arrows); (**b**) Two guide wires were introduced into the superior and inferior segments of the renal artery; (**c**) The suction catheter was introduced into the distal end of the superior occlusion segment; (**d**) Angiogram after suction catheter aspiration; (**e**) Angiogram after balloon expansion; (**f**) The guide wire was introduced into the inferior occlusion segment; (**g**) Angiogram after microcatheter injection of drugs; (**h**) Angiogram showing restoration of flow to the superior and inferior branches of the left renal artery (arrows); (**i**) Amplification of upper insert (solid line square) in **a**; (**j**) Amplification of lower insert (dash line square) in **h**; (**k**) Amplification of upper insert (solid line square) in **h**; (**l**) Amplification of lower insert (dash line square) in **h**

Routine laboratory tests showed normal complete blood count, liver function tests, amylase, lipase, and thyroid function tests. Creatinine was normal at 56 μmol/L (normal range, 40–130 μmol/L) with mildly elevated blood urea nitrogen (7.29 mmol/L [normal range, 2.3–7.1 mmol/L]). Urinalysis was negative for proteinuria, infection, and hematuria. Fecal occult blood test was negative. Both lactate dehydrogenase (LDH) and alpha-hydroxybutyrate dehydrogenase (α-HBDH) were elevated (283 U/l [normal range, 135–225 U/l] and 239 U/l [normal range, 76–195 U/l], respectively). Bedside abdominal and renal vascular Doppler ultrasound were normal. Due to persistent abdominal pain of unclear etiology, a contrast-enhanced CT scan was then performed, revealing renal infarction of the left kidney (Fig. 1A). Coronary (data not shown) and aortic angiograms (Fig. 1B) were negative for coronary stenosis and aortic dissection. Given the CT findings and unresolved abdominal pain, a renal angiogram was performed and showed distal occlusion of both superior and inferior segments of the left renal artery (Fig. 2a). Next, attention was paid to the left renal artery. Two guide wires were introduced into the left renal superior and inferior segments (Fig. 2b), and the Pt wire was then passed through the distal end of the occluded superior artery. A 5 × 4 mm thrombus was aspirated by a suction catheter and locally inflated using a Maverick 1.5 × 15 mm balloon, and 0.1 mg nitroglycerin and 1 mg diltiazem were injected through the microcatheter (Fig. 2c–g). Blood flow restoration to both superior and inferior segments of the renal artery and vessel potencies were confirmed by angiography after the aforementioned interventions (Fig. 2h), which is more clearly observed in the amplification of the images for renal angiograms after the surgery (Fig. 2k and l, arrow) compared with before the surgery (Fig. 2i and j, arrow). Abdominal symptoms resolved 6 hours after the operation. LDH (230 U/L) and α-HBDH (216 U/L) decreased and eventually normalized by postoperative day 5. He was continued on all home medications and started on warfarin post-procedure with an international normalized ratio (INR) goal of 2–3. The patient was discharged on triple therapy with aspirin (100 mg), clopidogrel (75 mg), and warfarin (3.125 mg) for 1 month, followed by clopidogrel and warfarin for a further 6 months. At 6 months follow-up, the patient was symptom-free and renal function remained normal (creatinine 74 μmol/L [normal range, 57–111 μmol/L]). Repeat angiography was not performed at follow-up to avoid additional radiation exposure given low suspicion for persistent or recurrent renal infarction.

## Discussion

We present a case of ARI caused by AF, where the patient presented with nonspecific abdominal pain and unremarkable laboratory markers. The ARI was confirmed with contrast-enhanced CT, and subsequently treated successfully with a combination of local thrombus aspiration, angioplasty, and local vasodilator therapies (nitroglycerin and diltiazem). The patient was then discharged on triple therapy with aspirin, clopidogrel, and warfarin for 1 month, followed by clopidogrel and warfarin for 6 months. At 6 months follow-up, the patient remained symptom free, with normal renal functions and urinalysis on laboratory testing. Cases of acute renal infarction have been previously reported, however, interventions varied. Local injection of vasodilators such as nitroglycerin and diltiazem are common vasodilator cocktails used in coronary angiogram but rarely used for the treatment of ARI. Our case demonstrates that a combination intervention of local thrombus aspiration, angioplasty, and vasodilator therapy results in a favorable long-term outcome.

Incidence of renal infarction is only 0.007%, and is most commonly caused by embolism from AF [1, 8–10]. Due to the rarity of ARI, there are only few reports on the effective therapeutic strategies to prevent kidney injury [3]. ARI often leads to partial or complete renal injury [10–12]; therefore, prompt diagnosis and intervention are essential to prevent permanent ischemic damage [13]. The key to ARI treatment is to restore renal blood flow as soon as possible, and the treatment mainly includes anticoagulation, thrombolysis, and surgery. Anticoagulation therapy can prevent further embolic events, and patients with renal artery embolism should be treated with anticoagulation once they are diagnosed. Even though treatment with systemic anticoagulation alone is commonly used, it often results in treatment failure and does not alleviate abdominal pain [1]. Thrombolytic therapy includes systemic intravenous thrombolysis and selective intra-arterial thrombolysis. Systemic thrombolytics are associated with an increased risk of bleeding. Compared with systemic thrombolysis, selective intra-arterial thrombolysis has a rapid onset of action and a lower risk of systemic bleeding. For patients with severe thromboembolism and relatively long embolization time, direct thrombolysis may not be effective, and thrombolysis can be performed after thrombus aspiration through an interventional catheter. Various thrombolytic methods have been reported including local injection of ornithokinin, tissue plasminogen activator, and other thrombolytic drugs, followed by thrombus aspiration, or thrombus aspiration followed by thrombolysis, with possible angioplasty [14]. Despite aggressive treatment, renal infarction can lead to acute kidney injury, new-onset eGFR < 60 mL/minute/1.73 m^2^ and death. Complete revascularization with stents has also been reported [6], but long-term antiplatelet therapy and the size of the culprit artery limit the use of this method. Better treatments are urgently needed [15].

In this case, our intervention quickly revascularized the occluded vessels, prevented permanent kidney damage, improved abdominal pain, and had minimal side effects. Local thrombus aspiration, balloon dilatation, and injection of vasodilators are not novel procedures, but the positive outcome in our case provides a promising new therapeutic intervention approach for AF-induced ARI while substantially reducing the risk of bleeding.

Additionally, laboratory tests are nonspecific in ARI. Previous reports suggested that elevation of LDH and C-reactive protein may be related to prognosis [16]. In our case, serum levels of LDH and α-HBDH were both elevated at the onset of symptoms and normalized after revascularization, suggesting that LDH and α-HBDH can potentially be used as markers for the diagnosis and treatment of ARI.

## Conclusions

In summary, we report an uncommon cause of abdominal pain, AF-induced ARI, that was treated successfully with a combination of local thrombus aspiration, angioplasty, and infusion of vasodilators, resulting in normal renal function and absence of recurrent infarction. Our case also demonstrated that elevation of LDH and α-HBDH can be used as diagnostic characteristics for ARI and correlate with disease state.

## Data Availability

The patient data for the current study are not publicly accessible by local health research ethics protocols; however, the data with de-identification may be available from the corresponding author.

## References

[1] Oh YK, Yang CW, Kim YL, *et al*. Clinical characteristics, and outcomes of renal infarction. Am J Kidney Dis. 2016;67(2):243–50. 10.1053/j.ajkd.2015.09.019. 26545635

[2] Silverberg D, Menes T, Rimon U, Salomon O, Halak M. Acute renal artery occlusion: presentation, treatment, and outcome. J Vasc Surg. 2016;64(4):1026–32. 10.1016/j.jvs.2016.04.043. 27345378

[3] Komolafe B, Dishmon D, Sultan W, Khouzam RN. Successful aspiration and rheolytic thrombectomy of a renal artery infarct and review of the current literature. Can J Cardiol. 2012;28(6):760. 10.1016/j.cjca.2012.06.020. 22906801

[4] Yousif A, Samannan R, Abu-Fadel M. Unilateral acute renal artery embolism: an index case of successful mechanical aspiration thrombectomy with use of penumbra indigo aspiration system and a review of the literature. Vasc Endovascular Surg. 2018;52(5):391–4. 10.1177/1538574418764052. 29554859

[5] Law Y, Chan YC, Cheng SW. Aspiration thrombectomy of acute atrial fibrillation-related renal artery thromboembolism in a patient with horseshoe kidney. Ann Vasc Surg. 2016;36(289):e5-89. 10.1016/j.avsg.2016.02.030. 27354320

[6] Tan TW, Bohannon WT, Mattos MA, Hodgson KJ, Farber A. Percutaneous mechanical thrombectomy and pharmacologic thrombolysis for renal artery embolism: case report and review of endovascular treatment. Int J Angiol. 2011;20(2):111–6. 10.1055/s-0031-1279682. PMC3331639 22654475

[7] Nagasawa T, Matsuda K, Takeuchi Y, *et al*. A case series of acute renal infarction at a single center in Japan. Clin Exp Nephrol. 2016;20(3):411–5. 10.1007/s10157-015-1168-1. 26377692

[8] Bourgault M, Grimbert P, Verret C, *et al*. Acute renal infarction: a case series. Clin J Am Soc Nephrol. 2013;8(3):392–8. 10.2215/CJN.05570612. PMC3586969 23204242

[9] Faucon AL, Bobrie G, Jannot AS, *et al*. Cause of renal infarction: a retrospective analysis of 186 consecutive cases. J Hypertens. 2018;36(3):634–40. 10.1097/HJH.0000000000001588. 29045340

[10] Garcia-Garcia A, Demelo-Rodriguez P, Ordieres-Ortega L, *et al*. Idiopathic versus provoked renal infarction: characteristics and long-term follow-up of a cohort of patients in a tertiary hospital. Kidney Blood Press Res. 2019;44(6):1432–40. 10.1159/000503425. 31683270

[11] Kwon JH, Oh BJ, Ha SO, Kim DY, Do HH. Renal complications in patients with renal infarction: prevalence and risk factors. Kidney Blood Press Res. 2016;41(6):865–72. 10.1159/000452589. 27871081

[12] Caravaca-Fontan F, Pampa Saico S, Elias Trivino S, *et al*. Acute renal infarction: clinical characteristics and prognostic factors. Nefrologia. 2016;36(2):141–8. 10.1016/j.nefro.2015.09.015. 26698927

[13] De Matteis G, Cutillo F, Contegiacomo A, Santoliquido A, Gambassi G. An uncommon cause of acute flank pain: renal infarction. Intern Emerg Med. 2019;14(3):485–8. 10.1007/s11739-018-2001-0. 30523496

[14] Ruzsa Z, Vamosi Z, Berta B, Nemes B, Toth K, Kovacs N, Zima E, Becker D, Merkely B. Catheter directed thrombolytic therapy and aspiration thrombectomy in intermediate pulmonary embolism with long term results. Cardiol J. 2020;27(4):363–75. 10.5603/CJ.a2020.0060. PMC8015998 32329040

[15] Oh YK, Yang CW, Kim YL, *et al*. Clinical characteristics and outcomes of renal infarction. Am J Kidney Dis. 2016;67(2):243–50. 10.1053/j.ajkd.2015.09.019. 26545635

[16] Yang J, Lee JY, Na YJ, *et al*. Risk factors and outcomes of acute renal infarction. Kidney Res Clin Pract. 2016;35(2):90–5. 10.1016/j.krcp.2016.04.001. PMC4919718 27366663

